# Successful Reboot of High-Performance Sporting Activities by Japanese National Women’s Handball Team in Tokyo, 2020 during the COVID-19 Pandemic: An Initiative Using the Japan Sports–Cyber Physical System (JS–CPS) of the Sports Research Innovation Project (SRIP)

**DOI:** 10.3390/ijerph18189865

**Published:** 2021-09-18

**Authors:** Issei Ogasawara, Shigeto Hamaguchi, Ryosuke Hasegawa, Yukihiro Akeda, Naoki Ota, Gajanan S. Revankar, Shoji Konda, Takashi Taguchi, Toshiya Takanouchi, Kojiro Imoto, Nobukazu Okimoto, Katsuhiko Sakuma, Akira Uchiyama, Keita Yamasaki, Teruo Higashino, Kazunori Tomono, Ken Nakata

**Affiliations:** 1 Department of Health and Sport Sciences, Graduate School of Medicine, Osaka University, Osaka 5650871, Japan; ogasawaraissei@hss.osaka-u.ac.jp (I.O.); revankar@neurol.med.osaka-u.ac.jp (G.S.R.); skonda@caos.med.osaka-u.ac.jp (S.K.); yamasaki@gim.med.osaka-u.ac.jp (K.Y.); 2 Department of Infection Control and Prevention, Graduate School of Medicine, Osaka University, Osaka 5650871, Japan; hamaguchi@hp-infect.med.osaka-u.ac.jp (S.H.); akeda@biken.osaka-u.ac.jp (Y.A.); naota@hp-infect.med.osaka-u.ac.jp (N.O.); tomono@hp-infect.med.osaka-u.ac.jp (K.T.); 3 Division of Infection Control and Prevention, Osaka University Hospital, Osaka 5650871, Japan; 4 Department of Information Networking, Graduate School of Information Science and Technology, Osaka University, Osaka 5650871, Japan; r-hasegawa@ist.osaka-u.ac.jp; 5 Japan-Thailand Research Collaboration Centre on Emerging and Re-Emerging Infections, Research Institute for Microbial Diseases, Osaka University, Osaka 5650871, Japan; uchiyama@ist.osaka-u.ac.jp (A.U.); higashino@ist.osaka-u.ac.jp (T.H.); 6 Institute for Transdisciplinary Graduate Degree Programs, Osaka University, Osaka 5650871, Japan; 7 Japan Handball Association, Tokyo 1600013, Japan; t-taguchi@japan-handball.jp (T.T.); toshiya_takanouchi@t-function.co.jp (T.T.); patagonia.imoto@gmail.com (K.I.); noboki4@yahoo.co.jp (N.O.); Katsu.Sakuma@gmail.com (K.S.); 8 Faculty of Economics and Information, Gifu Shotoku Gakuen University, Gifu 5008288, Japan; 9 T-function Inc., Tokyo 1560042, Japan; 10 Department of Orthopedic Surgery, Japanese Red Cross Kumamoto Hospital, Kumamoto 8618520, Japan; 11 Medical Corporation Okimoto Clinic, Hiroshima 7340304, Japan; 12 Department of Rehabilitation, Medical Corporation Yamabe-Kai, Kumamoto Seijo Hospital, Kumamoto 8618072, Japan

**Keywords:** handball, COVID-19, infection control, performance enhancement, stay active, proximity, heart rate, Tokyo 2020, physical activity, Japanese athletes

## Abstract

The COVID-19 pandemic has negatively impacted sporting activities across the world. However, practical training strategies for athletes to reduce the risk of infection during the pandemic have not been definitively studied. The purpose of this report was to provide an overview of the challenges we encountered during the reboot of high-performance sporting activities of the Japanese national handball team during the 3rd wave of the COVID-19 pandemic in Tokyo, Japan. Twenty-nine Japanese national women’s handball players and 24 staff participated in the study. To initiate the reboot of their first training camp after COVID-19 stay-home social policy, we conducted: web-based health-monitoring, SARS-CoV-2 screening with polymerase chain reaction (PCR) tests, real-time automated quantitative monitoring of social distancing on court using a moving image-based artificial intelligence (AI) algorithm, physical intensity evaluation with wearable heart rate (HR) and acceleration sensors, and a self-reported online questionnaire. The training camp was conducted successfully with no COVID-19 infections. The web-based health monitoring and the frequent PCR testing with short turnaround times contributed remarkably to early detection of athletes’ health problems and to risk screening. During handball, AI-based on-court social-distance monitoring revealed key time-dependent spatial metrics to define player-to-player proximity. This information facilitated appropriate on- and off-game distancing behavior for teammates. Athletes regularly achieved around 80% of maximum HR during training, indicating anticipated improvements in achieving their physical intensities. Self-reported questionnaires related to the COVID management in the training camp revealed a sense of security among the athletes that allowed them to focus singularly on their training. The challenges discussed herein provided us considerable knowledge about creating and managing a safe environment for high-performing athletes in the COVID-19 pandemic via the Japan Sports–Cyber Physical System (JS–CPS) of the Sports Research Innovation Project (SRIP, Japan Sports Agency, Tokyo, Japan). This report is envisioned to provide informed decisions to coaches, trainers, policymakers from the sports federations in creating targeted, infection-free, sporting and training environments.

## 1. Introduction

The COVID-19 outbreak and the associated home confinement strategy have significantly altered not only the daily lives of the general public, but also the sporting activities of athletes [1,2]. The negative effects of activity restrictions on athletes ranged widely, from reducing physical activity levels [3] to interfering with psychological stability in their lives [4,5] to introducing career path concerns [6], and athletes were required to respond appropriately to these matters. In particular, the postponement of the Tokyo Olympic/Paralympic Summer Games 2020 severely impacted the global sporting schedules, compelling athletes and coaches of sports federations to quickly determine and adopt innovative strategies to augment performance while controlling the spread of COVID-19 infections.

With viral transmission mechanisms becoming clearer, preventive measures have become the mainstay of addressing individual-level risk control [7]. However, these actions are sometimes impractical when dealing with elite athletes. Wearing a mask during high-intensity sports may interfere with normal ventilation and lead to hypoxia [8]. Expecting social distancing in a sport that involves physical contact is inherently futile. Therefore, it is imperative to establish a novel, state-of-the-art support protocol that enhances the performance of athletes during the COVID-19 pandemic. To this end, we studied the modalities of applying cutting-edge technology in the current scenario and the benefits of such approaches in sports medical sciences.

Handball is an indoor, contact sport in which two teams pass a ball using their hands, aiming to throw at the goal of the opponent team. The sport requires an intermittent repetition of aerobic and anaerobic demands during games, resulting in a high respiratory rate in such athletes [9]. The WHO considers such indoor contact ball games as high-risk sporting activities for virus spread [10]. Under the aforementioned playing conditions, including an infected player in a team is likely to facilitate the transmission of the virus and cause cluster outbreaks. Currently, for athlete training, by limiting contact with the outside world, “bubble”-style training camps are known to reduce the risk of infections, thereby allowing safe training practices with teammates and leading to high compliance [11]. Considering the high success rates of “bubble” camps [12] and a fundamental understanding of infection control for COVID-19, we established a high-impact, safe training/on-court game environment incorporating novel risk-monitoring methodologies to improve athlete performance in contact sports.

The conceptualized control strategies/guidelines in elite sports and sporting events were widely announced in 2020 [13,14,15]. Specifically, with respect to handball training/playing environments during the COVID era, research is limited. Because of the paucity of literature in this field, we present here a report on the national-level handball team that participated in the Tokyo Olympics with these guidelines in consideration. The main purpose was to provide an overview of the challenges encountered during the reboot of high-performance sporting activities of the handball team during the third wave of the COVID-19 pandemic in Tokyo, Japan. Another aim of this work was to understand the requirements of the athlete training camp and provide awareness that coaches, trainers, and policy makers from the national sports federations can use. Actions that formed the foundation of this reboot were: (1) web-based health-monitoring; (2) SARS-CoV-2 screening with frequent polymerase chain reaction (PCR) tests; (3) real-time monitoring of social distancing on court using a video-based artificial intelligence (AI) algorithm; and (4) heart rate and physical intensity evaluation using wearable inertia sensors.

## 2. Materials and Methods

### 2.1. Participant Information

We evaluated 29 Japanese national women’s handball players and 24 staff members (one head coach, five assistant coaches, three athletic trainers, one translator, three analysts, three nutrition specialists, and eight medical doctors). This research was performed in accordance with all relevant guidelines and regulations as well as the Declaration of Helsinki. The purpose of the study was explained to all participants using documents authorized by the JHA and the ethics committee of the Osaka University Hospital (19537-2). Written informed consent was obtained from all participants. Every athlete was a domestic player at the time of testing. Their home teams were from Kumamoto, Kagoshima, Ishikawa, Osaka, Mie, Tokyo, Ibaraki, and Hiroshima prefectures and travelled to Tokyo on 24 November 2020 after confirming COVID-19 negative status with precamp PCR testing as described below. None of the athletes and staff members were vaccinated before the beginning of the training camp.

### 2.2. History of Training Restrictions Due to Japan’s State of Emergency before Rebooting the National Team’s Training Camp

On 24 March 2020, the Japanese government announced the postponement of the Tokyo Olympics and Paralympics, followed by the declaration of a state of emergency on 7 April. As a result, the Japanese national handball team canceled all training schedules from April to August, specifically, all domestic competitions, three domestic training camps (April, May, and July), and an international training trip in three European countries (May–June). During this period, the national team players continued to train themselves under restricted conditions, basically at their home teams, with some discrepancies among the teams. The regular handball training gathering all team members was basically canceled from April to June 2020. Instead, team members practiced handball in small groups of three to five players. They also continued individual endurance, agility, and resistance training. The team physical coach remotely instructed the individualized training during the period of restricted activity. After the Japanese government declared the end of the state of emergency on 25 May, each home team resumed regular handball practice in stages until the end of July. Therefore, by the time the national team’s first training camp after COVID-19 began on 24 November 2020, each national team player had been conducting regular handball practice for about four months.

### 2.3. Details of the First Domestic Training Camp after Japan’s COVID-19 State of Emergency and “Stay-Home” Social Policy

The training camp was held at the Ajinomoto National Training Center (ANTC) in Tokyo. The ANTC is a national sports complex designated for high performance sports. This was the first time a training camp of this scale was organized after Japan’s COVID-19 state of emergency and “stay-home” social policy. The 2-week camp started on 24 November 2020 and was completed on 8 December 2020. The reboot of the camp was approved by the Japan Olympic Committee (JOC), the JSA, and the Japanese Ministry of Health, Labor, and Welfare (MHLW) based on well planned regulations within the training halls and emergency protocols prepared by the JHA in advance. The health requirements to participate in the camp were the lack of a fever above 37.5 °C in the past two weeks and a negative PCR test conducted four days prior to the camp admission. On day 1, all players and staff were briefed by the JHA with reference to the protocols for team compliance during the training camp. The training plan was designed by the head coach to enhance athletes’ physical capabilities, handball skills, and tactics. It consisted of twenty training sessions including handball practice, training matches, weight training, functional movement training, physical performance tests, and medical checkups.

### 2.4. Scientific Monitoring

The scientific monitoring was jointly carried out by the JHA, the Osaka University, and the Japan Sports Agency (JSA) leading the activity of the JS–CPS (Japan Sports–Cyber Physical System) of the Sports Research Innovation Project (SRIP, JSA, Japan).

#### 2.4.1. Web-Based Health Monitoring

All athletes documented their body temperature, body weight, and subjective health conditions every day via a web-based system (One Tap Sports, Euphoria Co., Ltd., Tokyo, Japan). In early morning, athletes measured their axillary temperatures with their thermometers in bed immediately after waking up. Monitoring duration was from 10 November to 22 December 2020 (2 weeks before and after the training camp). When body temperatures above 37.5 °C were reported, the system alerted the medical staff via email regarding the corresponding athlete’s status.

#### 2.4.2. Screening of SARS-CoV-2 with Frequent PCR Tests

A total of 10 PCR testing sessions (364 samples), which included precamp screening and in- and postcamp inspection, were performed. Saliva samples were examined by a SARS-CoV-2 Direct Detection RT-qPCR Kit (Takara Bio, Otsu, Shiga, Japan) using a LightCycler 96 (Roche Molecular Systems, Branchburg, NJ, USA) for the detection of SARS-CoV-2 according to the methods described by the manufacturers. The specificity (100% = 10/10 positive samples detected) and the sensitivity (100% = 15/15 negative samples detected) of the SARS-CoV-2 Direct Detection RT-qPCR Kit were confirmed by the National Institute of Infectious Diseases (NIID), Japan [https://www.niid.go.jp/niid/images/lab-manual/2019-nCoV-17-current.pdf (Japanese document) accessed on 28 August 2021].

- Precamp screening and postcamp verification.
  Each individual sampled their saliva (1 mL) at their residence under the online guidance of the researcher (I.O) on 19 November and 9 December, thereby providing pre- and postcamp samples, respectively. The centrifuge tube (primary packaging) containing saliva was wrapped with an absorbent sheet and tightly sealed in the biopouch (secondary packaging). The sample was then placed in a bio-mailer box (tertiary packaging) and sealed with a security sticker. Finally, the bio-mailer box was put into a quaternary packaging box. This box was then transported under the regulation of MHWL (Category B, UN3373) to the Group of Infection Control and Prevention (G-ICP) in Osaka University Hospital via Japan Post Co., Ltd., Tokyo, Japan. The results of pre- and postcamp PCR screening (all negative) were reported to JHA via email.
- SARS-CoV-2 PCR testing protocol in the camp at ANTC.
  During the camp period (from 24 November to 8 December), SARS-CoV-2 PCR testing was conducted every Monday, Wednesday, and Friday (ideally every 48 h, excluding weekends) [16]. On the “test day”, each athlete and staff sampled their saliva around 8:00 am. The samples were then immediately transported to the semi-onsite PCR center held in the Toho University Medical School (about 1.0 h from ANTC) and tested by the staff of the Osaka University ICP team. The mean sample-to-answer (turnaround) time was 4.2 h (including 1.0 h of sample transportation time); therefore. the team staff could announce the results of the PCR tests at around 13:00 on every testing day, prior to the start of the afternoon training session (~15:30).

#### 2.4.3. Real-Time Automated Quantitative Monitoring of Social Distancing with Motion Image-Based AI System

**Figure 1:**
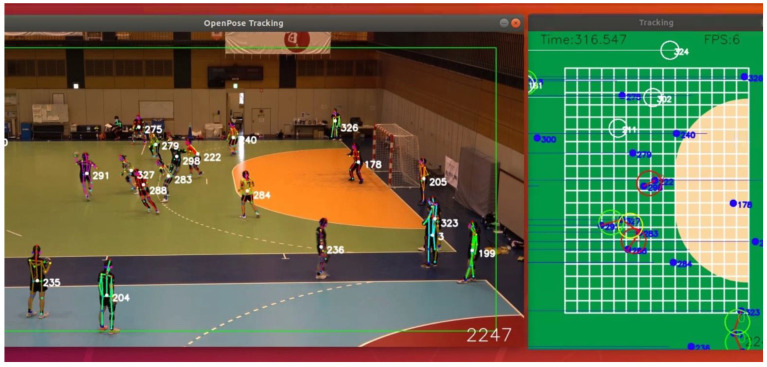
Real-time automated quantitative monitoring of social distancing with a motion image-based AI system, shown on Ubuntu 18.04.5 LST. This system identified participant positions using a function on OpenPose [17]. The distance between the players was calibrated based on known court geometry.

The degree of social distancing on the handball court was monitored by a custom-made AI system (Figure 1). The system calculated interpersonal distance in real time using motion images and scored the degree of proximity (Japanese Patent Application No.2020-123769). When the system identified two athletes within a distance of 2.0 m, it assigned a unique proximity ID to that pair by marking a white circle in the video image and added one proximity point every second when this distance was maintained continuously. The color of the circle turned green (2 s), yellow (3 s), and red (>4 s) depending on the continuous time of the close contact (Figure 1). This system gave real-time visual feedback on the degree of on-court proximity with the color of the circles to the coaches and athletes. Furthermore, the system stored the (x, y) position coordinates of all pairs of athletes at <2.0 m distance within the 1/30 s timestamp information in a character-separated value (CSV) file for subsequent offline analysis.

#### 2.4.4. Heart Rate and Activity Monitoring with a Wearable Sensor Device

To quantify the intensity of physical activity, we used a wearable sensor that measured heart rate (HR) and truncal acceleration. The purpose of this recording was to evaluate whether the physical demands on each player were high enough to enhance their performance. Athletes wore electrodes sewn onto sports bras with a small wearable sensor (SS-ECG, Teijin Frontier Sensing Co., Ltd., Fukuoka, Japan) to record the electrocardiogram (ECG) and three-dimensional acceleration of the torso at 200 Hz. Truncal (3D) accelerometer data was used only to define the active phases during the training matches. The HR measurements were performed during a 3000 m high-intensity endurance time trial test (30 November) and during eight training matches (15 min each, 5–6 December). Given the physically demanding nature of the 3000 m time trial test, we used the HR max value obtained during the 3000 m run as a surrogate marker to evaluate the physical intensity during handball matches.

### 2.5. Data Analysis

From the web-based body temperature monitoring data, daily changes in the body temperature of all athletes and staff members were graphed. The results of the self-reported online questionnaire regarding the impression and general impact of the current PCR testing were visualized as a five-point Likert scale.

For the social distancing AI monitoring data, the total sum of the proximity points for all proximity ID pairs was normalized by the number of associated athletes and defined as the cumulative proximity score. The cumulative proximity score was normalized by two separate unit time (UT) scales to consider the effect of respiratory rate (RR) on physical activity, since the risk of virus spread is theoretically proportional to RR, which is increased by physical activity [18,19]. The UT roughly assumed a time needed for one respiratory cycle and was calculated based on the measured average HR at rest (about 60 bpm) and during a handball match (about 140 bpm) from the participant. Referring to Scholkmann et al. [19], the ratio between the time needed for one heartbeat and that for one respiratory cycle is about 1:4. Thus, the UT (time needed for one respiratory cycle) was calculated as 60 s/60 bpm × 4 = 4 s while at rest and 60 s/140 bpm × 4 = 2 s while in training. The total time that was maintained continuously for each proximity ID served as a metric for proximity duration. With the aforementioned metrics in place, we evaluated two training matches (each lasting 15 min) held on 6 December 2020. Additionally, midway during the camp (day 5), the system identified that the stretching time (pregame) contributed to high proximity scores. We intervened and provided feedback to the teams to disperse during stretching time on the remaining days. Proximity scores are therefore described under results before (up to day 5) and after (day 6 onwards) trainers’ feedback. To visualize high-proximity locations on the handball court, a two-dimensional frequency histogram was visualized using the (x, y) position coordinate data of every proximity ID pair recorded during two training matches and stretching exercises. The spatial resolution for calculating two-dimensional histogram was set as 0.3 × 0.3 m squares. The visualization was performed with the Matlab function “densityplot”. Furthermore, based on the timestamp information, the continuous times of all proximity ID were calculated, and the frequency density of the proximity duration was compared between handball matches and stretching.

Based on the R–R interval of the ECG signal recorded with the wearable sensor device, the HR data (bpm) for the 3000 m high-intensity endurance test and the eight training matches were calculated. The maximum HR value for each athlete observed during the 3000 m run test was used to normalize the HR value during handball matches. The 3D acceleration data were used to determine the effective playing time. The norm of the 3D acceleration signal $\left|a\right|=\sqrt{a_{x}^{2}+a_{y}^{2}+a_{z}^{2}}$ was calculated and high-pass filtered (second order zero-lag Butterworth digital filter, cutoff frequency = 0.03 Hz) to eliminate the effect of the gravitational acceleration. Then the rectified norm acceleration signal was low-pass filtered (second order zero-lag Butterworth digital filter, cutoff frequency = 0.5 Hz) to obtain a smooth signal. We assumed that an athlete was in play when their corresponding smoothed norm acceleration signal |a| was more than 0.3 G based on our preliminary analysis (data not shown). The effective HR data for the in-play period were therefore extracted where the smoothed norm acceleration signal |a| exceeded 0.3 G. Using the effective HR data, the percentage of total effective playing time spent in different HR zones (zone 1: <70%, zone 2: <85%, zone 3: <90%, zone 4: <95%, and zone 5: ≧95% of HR max) was calculated for each athlete. The segmentation of HR zones was based on the report by Manchado et al. [20], which evaluated the match performance of elite women’s handball teams far before the COVID-19 pandemic. Each HR zone percentage value was separately averaged for back players (BP), pivot players (PV), wing players (WG), and goalkeepers (GK), because the physical demands for each role were expected to vary.

All numerical processing was performed with Matlab R2020a and R v4.0.2. Data analyzed in this manuscript will be made available from the corresponding author upon reasonable request.

## 3. Results

Demographic information on the participants is detailed in Table 1.

The reporting rate of the web-based monitoring was 88.7% throughout the survey period (10 November to 22 December) and 96.6% during the camp (24 November to 8 December). Every athlete completed their web-based monitoring.

SARS-CoV-2 PCR testing history and results are summarized in Table 2. According to the results of precamp screening (all negative for SARS-CoV-2, informed via email) and confirmation of nonfebrile status (>37.5 °C) for two weeks prior the training camp, athletes were invited to the training camp by the JHA. In-camp PCR tests among all athletes were also negative.

**Figure 2:**
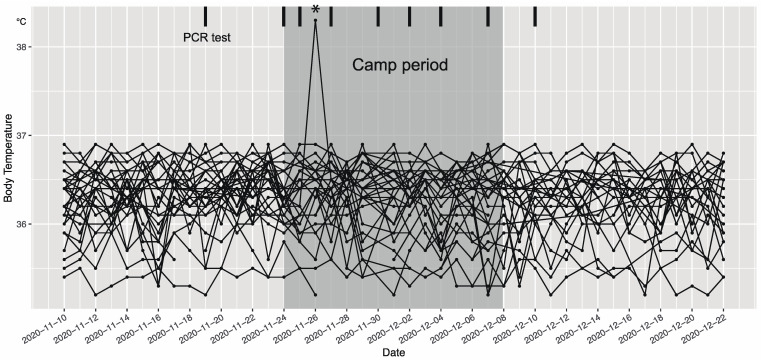
Body temperature chart line–scatter plot for all athletes. The darker grey shaded area is the camp period (15 days); thick vertical lines denote days on which PCR tests were performed; and the asterisk denotes the single febrile athlete recorded on day 3.

A summary overview of the temperature checks documented by the participants is graphed in Figure 2. As shown, a single athlete reported febrile status on day 3 (~38.5 °C). According to the emergency protocol created by the JHA, all athletes were temporarily quarantined until the PCR results were confirmed. An exclusive PCR test for this febrile athlete was performed. Three other athletes from the same home team were also tested immediately. Though this was not the regular “test day”, samples from these four athletes were dispatched at 10:00 and the results (all negative) arrived by 13:00. On confirmation of a negative result, all athletes except the febrile athlete restarted their training schedule on the same day. The sick athlete returned to training, after recuperation and three further negative PCR tests, on 3 December.

**Figure 3:**
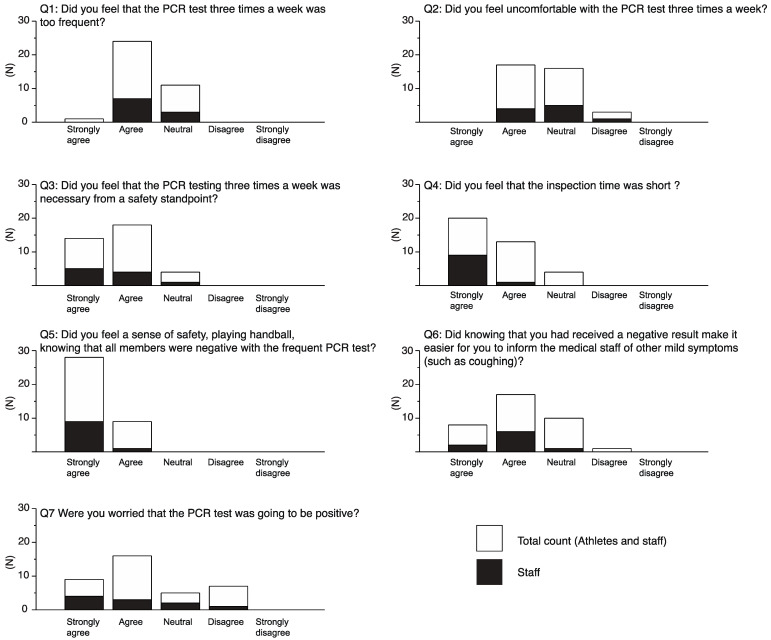
Results of online questionnaire regarding perception of PCR tests during the camp.

Results from the online questionnaire about infection test protocol were obtained from 27 athletes and 10 staff (Figure 3 Q1–Q7). Our survey suggested that most participants felt that three tests a week was too frequent (Q1) and were not compliant (Q2), but recognized the importance of high-frequency testing to maintain a safe training environment (Q3). The frequent testing with a short sample-to-answer time provided a strong sense of relief both to athletes and staff that helped them participate in the handball training sessions (Q4, Q5). Moreover, being aware of the negative results following every testing session increased their compliance in reporting minor symptoms (such as coughing) to the medical staff (Q6). Most participants felt anxious while waiting for the results on each test day (9 strongly agree and 16 agree); however, most appeared unconcerned about the PCR results (5 neutrals and 7 disagree, Q7).

**Figure 4:**
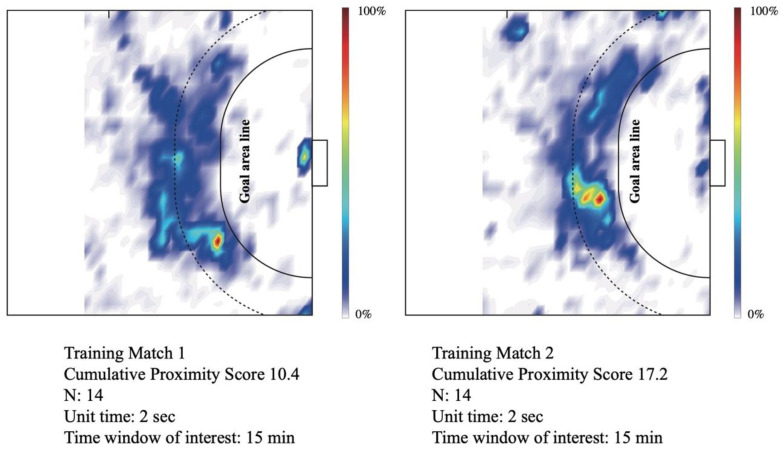
Heat map of area-specific proximity density on the handball court during training matches. The color map represents a 0 to 100% scaled value from areas where no proximity IDs were observed (0%, white) to areas where the most frequent proximity IDs were observed (100%, dark red) during the time window of interest.

**Figure 5:**
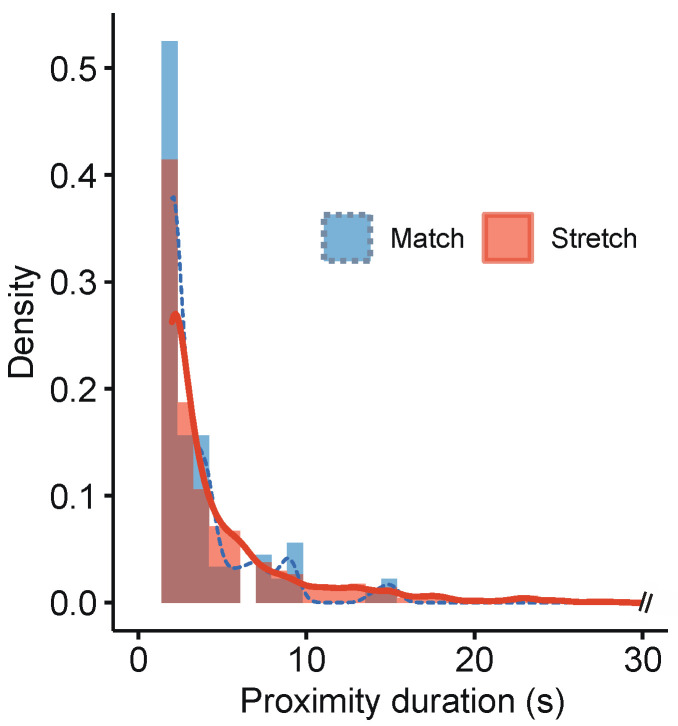
Density plot of the duration for each proximity ID during match time and stretching time.

Table 3 describes the situation-specific proximity scores gathered from real-time video-based AI monitoring. During the 15 min training matches, the cumulative proximity scores were 10.4 and 17.2. According to the Japanese guidelines [21], the definition of close contact is a distance within 1 m maintained for 15 min. The cumulative proximity score of this definition is 15 min × 60 s/2 s of UT/2 persons = 225 points. Therefore, it was found that the cumulative proximity scores during the 15 min training matches were lower than the Japanese definition of the close contact. Although a high proximity occurred along the goal-area line (Figure 4), the histogram of proximity duration for each proximity ID illustrated that during the match, the frequency of short-duration proximity IDs was high, while during stretching, the frequency of long-duration proximity IDs was high, because every player was dynamically moving during the games (Figure 5).

**Figure 6:**
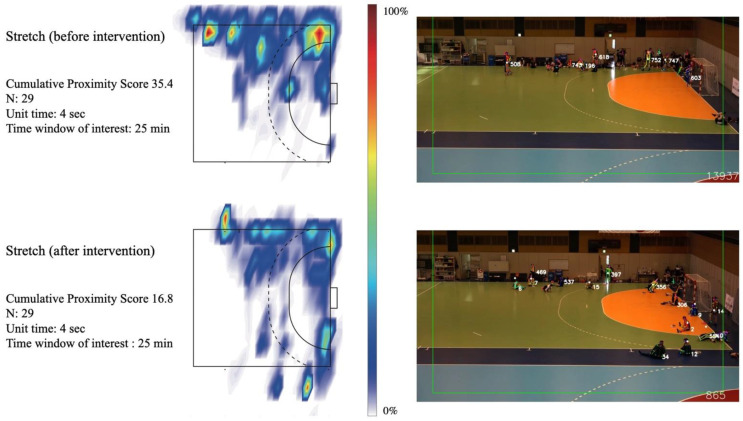
Transition of high-proximity distribution during stretching, before and after intervention. The color map represents a 0 to 100% scaled value where no proximity IDs observed (0%, white) to where the most frequently proximity IDs observed (100%, dark red) during the time window of interest.

We found that many athletes concentrated in a specific place during stretching time before practice (score = 35.4, averaged proximity duration = 3.9 s). After providing feedback on this issue to the team on 27 November (day 5), the location of the athletes during stretching became dispersed, and the proximity condition was clearly reduced thereafter (score = 16.8, averaged proximity duration = 2.5 s, Figure 6).

Evaluation results for HR and physical endurance are shown in Table 4. During the training matches (15 min × 4 set), the averaged maximum HR achieved by pivot players (PVs) = 194.9 bpm and by back players (BPs) = 191.9 bpm. These players have the most active roles on the court. In reference to the HR value measured during the 3000 m endurance run (a vigorous, exhausting running test), the maximum in-match %HR by BPs and PVs scored more than 100%. However, the averaged in-match %HR ranged between 74.7% (goalkeeper) and 84.5% (BPs).

The time spent in the different HR zones, expressed as percentage of the effective playing time, is shown in Table 5. The time spent in the HR zone higher than 85% of HRmax (Zone 3 to 5) was 63.6% for BPs, 60.7% for PVs, and 47.9% for WGs, respectively. GKs did not reach HR zones higher than 3; instead, they spent 97% of their total effective playing time in HR zones 1 and 2.

## 4. Discussion

We here present an overview of the challenges encountered by the JHA in developing a safe training environment and evaluate the outcome of risk management among high-performance athletes who rebooted their training despite the COVID-19 pandemic. With our comprehensive monitoring/feedback protocol, the Japanese national handball team was successful in completing the training camp with no COVID-19 positive cases and confirming their postlockdown physical level, which may help in planning their training requirements for future competitions.

### 4.1. Web-Based Health Monitoring

Continuous monitoring of an accurate health status is one of the first steps to minimize the spread of infection in a closed community such as a training camp. We observed no severe health problems related to the current training protocol despite one athlete presenting with fever (38.5 °C) on the third day of the training camp (Figure 2). With the help of a web-based system, the medical staff could immediately observe and identify health concerns when the system reported any issues. This also enabled us to manage all players’ health status while avoiding unnecessary contact between them.

It is important to mention that the responsibility of data entry rested solely on the athletes. It is possible that athletes were anxious of missing out on competitive opportunities, making them occasionally hesitant to disclose their health status. While it was commendable that one athlete reported her fever, it was important to build a sense of safety among other athletes as well. We surmised that crucial appraisal regarding infection control by the JHA and frequent negative PCR testing promoted a positive mental outlook that would have led athletes to genuinely report their true health condition (Figure 3 Q5, Q6). Regardless, web-based monitoring contributed positively to developing a conducive behavioral environment among the athletes.

### 4.2. SARS-CoV-2 PCR Testing and Feedback

Though we had no SARS-CoV-2-positive athletes during the entire duration of the camp, our frequent testing schedule (every 48 h or 72 h) enabled us to detect COVID-19-infectious athletes early, thereby minimizing the risk of virus spread. Larremore et al. suggested that repeated screening of susceptible but asymptomatic individuals could be used to limit transmission, and that the effectivity of screening therefore rests on accessibility, frequency, and sample-to-answer time [16]. The accessibility of the PCR test turned out to be crucial when an athlete was reportedly febrile. Even outside of the designated “testing days”, prompt PCR testing allowed us to confirm a negative test within 3 h of sample collection. Without the convenience of obtaining a PCR result for a febrile patient, a complete shut-down and quarantine of the training camp would have been necessary regardless of the actual infection status. Because we had a well-planned emergency protocol in force, we minimized the training loss, allowing the team to return to their regular training schedule on (the afternoon of) the same day.

The results of the online PCR survey highlighted the increased compliance of the athletes despite the high frequency of testing (Figure 3). While the participants were aware of the risk of transmission in a closed setup such as ours, the additional burden of testing did not, in fact, hamper their training sessions or their main objective in the camp (i.e., enhancing performance). Considering the effectivity of PCR testing in such scenarios, we recommend that for national training centers such as ANTC, an exclusive PCR center as a primary facility will be indispensable in the future. Furthermore, it would be desirable to establish a mass screening methodology that enables a low-cost on-court inspection to improve test accessibility.

### 4.3. Real-Time Automated Qualitative Monitoring of Social Distancing on the Handball Court Using a Video-Based AI Algorithm

Real-time quantitative monitoring using AI demonstrated the characteristics of the proximity status that occurred during on-court handball training. The quantitative visualization by the AI system showed that off-game situations such as stretching are more likely to result in significant proximity than in-game handball situations, and that these proximities can be alleviated by behavioral changes of athletes. In this way, the AI system was able to provide objective numerical scores to improve athlete behavior.

If the stretching time could be construed as a “control” environment, the in-game matches served as a “test” condition to compare the proximity status between the players. A continuous-time density plot revealed that the proximities during a handball match were not as long-lasting as it was during stretching (Figure 5). The cumulative proximity scores during handball matches were considerably lower when compared to those during stretching (Table 3). Because of the highly dynamic nature of the sport, it was difficult to conclude the proximity outcomes in matches, since none of the players were near enough for long enough for significant change in the readings. Conversely, we could interpret that despite participating in a body-contact sport, the athletes were never too close for long durations, which is always a risk for virus transmission. Despite handball being a contact sport, there appears to be low evidence of transmission to support limiting sporting activities [22]. Without excessive speculation, we suggest that if the camp is “bubbled” with players who have tested negative, the results of real-time AI monitoring systems can positively influence the decision to remove masks, allowing athletes to play handball normally.

### 4.4. Heart Rate and Physical Intensity Evaluation Using the Wearable Sensor

Since the primary aim of the training camp was to increase athlete performance, it was essential to quantify whether the physical intensity was demanding enough to fulfill this objective. Kniubaite et al. [23] reported that the averaged %HR of the elite Lithuanian female handball players during international competition ranged from 84.2% to 84.8%. Manchado et al. [20] reported that the averaged %HR of top-level Norwegian and German women’s teams was 86.5 ± 4.5% for field players. In comparison, the averaged %HR of 80.7–84.5% in Japanese players (BP, PV, and WG, Table 4) was slightly lower than that in prior reports [23,24]. Our HR zone analysis demonstrated that the accumulated time spent in the high HR zone (Zones 3 to 5, more than 85% of HR max) for field players ranged from 47.9% to 63.6% of total effective playing time; this performance was also slightly lower than those of top handball teams (65% of HR max) reported in previous literature [20]. Furthermore, the percentage of time spent in the low intensity zone (Zones 1 to 2, less than 85% of HR max) ranged from 35.8% (BP) to 51.7% (WG), which was obviously greater than that of top handball teams (34.4% of total match time). This discrepancy in physical intensity during match between the Japanese national handball team and the previous data [20,23,24] evaluated before the COVID-19 pandemic may be attributed to the prolonged activity restrictions experienced by the Japanese players before rebooting the training camp. Similar adverse effects of the COVID-19-related activity restriction on athletes’ cardiovascular performance have been reported in many sports [25,26,27]. Most of our athlete cohort resumed their regular handball training step-by-step after the end of state of emergency in Japan. However, by the start of the training camp in November 2020, their peak physical performance level had not been fully achieved. Online monitoring of physical intensity level is an essential approach to capture the gap between a team’s current status and that of other competitive teams.

### 4.5. Limitations

It is important to note that the sample size for our study was low (29 athletes and about 10 staff per test day). Although our presented methodologies could be optimum for small sample sizes, for large-scale screening (>1000 samples) in the future, individuals organizing the team should consider an optimal screening strategy in terms of cost, technician availability, testing facilities, and warranting test accuracy. As with any self-reported survey procedure, there is a possibility of self-reporting bias among the athletes [28]. With rigorous testing and evaluation conditions, we believe that the responses of athletes were reasonable. The video-based position detection is a vital technology for large indoor environments where GPS or other positioning systems do not work. Since the technology is new, more comparative data is needed to fully validate its efficacy for risk quantification measured via proximity scoring. In its current state, we have provided a tentative interpretation of what high/low cumulative proximity scores are to be expected for infection control. In addition to increasing sample sizes, it will be beneficial in the future to implement individual assessment features in addition to the current cumulative evaluation.

## 5. Conclusions

Aiding athletes in disrupting physical inactivity and normalizing their training routines during the pandemic was our central purpose, which led to the rebooting of high-performance sporting activities under the JS–CPS (Japan Sports–Cyber Physical System) and by the SRIP (Sports Research Innovation Project). The pursuit resulted in the successful management of a training schedule among handball players, providing a safe training environment for their handball practice within a stringent COVID-19 screening environment. Our efforts in this study are expected to set the foundation for future endeavors in standardizing training protocols and for scaling up to include for other team-based indoor sporting activities. Specifically, we believe that the short turnaround time for PCR testing is vital for the early identification and prioritization during the screening process. As an added benefit, the outcomes of this process also develop a sense of security among the athletes. Based on our results, an on-site, low-cost mass screening methodology will be beneficial to develop in the future. Using information technology, real-time quantification of health status and proximity metrics will be an indispensable tool to quantify the effect of risk management. These technological innovations developed under SRIP research can constitute a promising approach in building a safe environment to support athletic training during the pandemic.

## Figures and Tables

**Table 1:** Demographic information on the participants.

	Athletes	Staff
Number of participant (female, male)	29 (29, 0)	24 (4, 20)
Mean age (SD)	28.4 (4.1) y	40.2 (14.2) y
Mean height (SD)	163.8 (18.8) cm	171.2 (11.1) cm
Mean weight (SD)	66.7 (8.3) kg	70.7 (13.2) kg
Mean training hours per week during camp	18 h per week	
History of COVID-19 infection before camp	None	None
COVID-19 infections after camp	None	None

**Table 2:** PCR test history and results.

Date	Schedule	Samples: Athletes	Samples: Staff	Results	Sample-to-Answer Time *	Note
20 November	Precamp	28	21	Negative	—	All athletes and staff transported their saliva samples to Osaka University from their home teams. Based on these results, the JHA recruited all athletes and staff.
24 November	In-camp, start up	28	11	Negative	5.5 h	
25 November	In-camp, regular	28	10	Negative	3 h	
25 November	In-camp, *irregular*	1	0	Negative	3 h	One additional athlete was invited.
26 November	In-camp, *irregular*	4	0	Negative	3 h	One athlete recorded a fever on the morning of 26 November. This athlete and three other athletes from the same home team were tested for screening purposes.
27 November	In-camp, regular	29	12	Negative	5.5 h	
30 November	In-camp, regular	29	12	Negative	5 h	
2 December	In-camp, regular	28	11	Negative	5 h	One athlete retired because of injury.
4 December	In-camp, regular	28	10	Negative	4 h	
7 December	In-camp, regular	28	11	Negative	4 h	
10 December	Postcamp	29	6	Negative	—	All athletes and staff transported their saliva samples to Osaka University from their home teams. Based on these results, the JHA allowed them to return to their regular practices and jobs.

* Sample-to-answer time or turnaround time was the time from the sampling of athletes’ saliva to the arrival of the e-mail announcing the results. This time included both the sample transportation time (approximately 1.0 h) and the inspection time. Our PCR test center (Toho University Medical School) was 28 km away from the training hall (ANTC). It took approximately 1.0 h by a car.

**Table 3:** Situation specific proximity scores.

	Cumulative Proximity Score (UT Adjusted)	Unit Time (s)	Maximum Proximity Duration (s)	Averaged Proximity Duration (s)	Time Window of Interest (min)	Number of Athletes in Video ROI
Training Match 1	10.4	2	21	2.4	15	14
Training Match 2	17.2	2	31	2.5	15	14
Stretch (before feedback)	35.4	4	56	3.9	25	29
Stretch (after feedback)	16.8	4	60	2.5	25	29

Time window of interest is the duration of evaluated event. UT: unit time.

**Table 4:** Heart rates at rest, during 3000 m endurance run, and training matches.

Position	At Rest (SD)	3000 m Run (SD)	Max in Match (%3000 m)	Average in Match (%3000 m)
BP	58.8 (13.4)	186.6 (8.5)	191.9 (104.0%)	155.9 (84.5%)
PV	61.0 (4.2)	186.0 (10.7)	194.9 (105.1%)	144.9 (81.9%)
WG	64.9 (9.1)	184.0 (7.3)	182.7 (99.4%)	148.4 (80.7%)
GK	67.3 (6.4)	185.3 (8.5)	169.7 (87.5%)	144.9 (74.7%)

At rest: minimum HR during stretching time before warming up. 3000 m run: maximum HR during 3000 m high-intensity endurance test. Max in match: maximum HR during matches (15 min × 4 set). Average in match: averaged HR during matches (15 min × 4 set). BP: back player, PV: pivot player, WG: wing player, GK: goalkeeper.

**Table 5:** Percentage of effective playing time spent in the different HR zones.

	Zone 1 (%)	Zone 2 (%)	Zone 3 (%)	Zone 4 (%)	Zone 5 (%)
BP	16.5 ± 8.8	19.2 ± 9.1	24.7 ± 11.1	23.2 ± 11.8	15.7 ± 18.0
PV	22.3 ± 10.8	16.5 ± 5.3	22.2 ± 7.0	30.2 ± 5.7	8.3 ± 6.7
WG	18.5 ± 12.1	33.2 ± 12.5	26.8 ± 9.6	15.8 ± 9.2	5.3 ± 6.2
GK	44.4 ± 25.9	53.3 ± 24.1	2.3 ± 1.8	0.0 ± 0.0	0.0 ± 0.0

Zone 1: <70%, Zone 2: <85%, Zone 3: <90%, Zone 4: <95%, and Zone 5: ≧95%.

## Data Availability

Data analyzed in this manuscript will be made available from the corresponding author upon reasonable request.

## Abbreviations

SARS-CoV-2	Severe acute respiratory syndrome coronavirus 2
CPS	Cyber physical system
PCR	Polymerase chain reaction
AI	Artificial intelligence
JHA	Japan Handball Association
SRIP	Sports Research Innovation Project, Osaka University
ANTC	Ajinomoto National Training Center
JSA	Japan Sports Agency
JOC	Japan Olympic Committee
MHWL	Japanese Ministry of Health, Labor, and Welfare
G-ICP	Group of Infection Control and Prevention, Osaka University
